# Supplementary material for: Endotyping-informed therapy for patients with chest pain and no obstructive coronary artery disease: a randomized trial
Source: Nat Med. 2025 Nov 10;32(1):332–41. doi: 10.1038/s41591-025-04044-4 (PMC12823439; doi:10.1038/s41591-025-04044-4)
Supplement: Supplementary file 1 — Supplementary Tables 1–7, Figs. 1–10 and clinician and patient guidance letters by diagnosis. [file 41591_2025_4044_MOESM1_ESM.pdf]

nature medicine

Supplementary information https://doi.org/10.1038/s41591-025-04044-4

Endotyping-informed therapy for patients with chest pain and no obstructive coronary artery disease: a randomized trial

In the format provided by the authors and unedited

Supplementary Tables

Table S1. Invasive Coronary Angiography in the Randomized Population.

	All<br>N=250	Control<br>N=126	Intervention<br>N=124
Coronary angiogram			
Right coronary artery stenosis severity, n (%)			
0%	221 (88.4%)	114 (90.5%)	107 (86.3%)
1-24%	15 (6.0%)	5 (4.0%)	10 (8.1%)
25-49%	13 (5.2%)	7 (5.6%)	6 (4.8%)
50-69%	1 (0.4%)	0 (0.0%)	1 (0.8%)
70-99%	0 (0.0%)	0 (0.0%)	0 (0.0%)
100%	0 (0.0%)	0 (0.0%)	0 (0.0%)
Left main coronary artery stenosis severity, n (%)			
0%	247 (98.8%)	124 (98.4%)	123 (99.2%)
1-24%	2 (0.8%)	2 (1.6%)	0 (0.0%)
25-49%	1 (0.4%)	0 (0.0%)	1 (0.8%)
50-69%	0 (0.0%)	0 (0.0%)	0 (0.0%)
70-99%	0 (0.0%)	0 (0.0%)	0 (0.0%)
100%	0 (0.0%)	0 (0.0%)	0 (0.0%)
Left anterior descending coronary artery stenosis severity, n (%)			
0%	202 (80.8%)	100 (79.4%)	102 (82.3%)
1-24%	20 (8.0%)	10 (7.9%)	10 (8.1%)
25-49%	20 (8.0%)	10 (7.9%)	10 (8.1%)
50-69%	8 (3.2%)	6 (4.8%)	2 (1.6%)
70-99%	0 (0.0%)	0 (0.0%)	0 (0.0%)
100%	0 (0.0%)	0 (0.0%)	0 (0.0%)
Circumflex stenosis severity, n (%)			
0%	231 (92.4%)	117 (92.9%)	114 (91.9%)
1-24%	6 (2.4%)	4 (3.2%)	2 (1.6%)
25-49%	9 (3.6%)	4 (3.2%)	5 (4.0%)
50-69%	4 (1.6%)	1 (0.8%)	3 (2.4%)
70-99%	0 (0.0%)	0 (0.0%)	0 (0.0%)
100%	0 (0.0%)	0 (0.0%)	0 (0.0%)
Angiographically normal, n (%)	190 (76.0%)	94 (74.6%)	96 (77.4%)
Coronary dominance, n (%)			
Left	20 (8.0%)	13 (10.3%)	7 (5.6%)
Right	230 (92.0%)	113 (89.7%)	117 (94.4%)
Invasive coronary function test			
Any coronary function test performed, n (%)	21 (8.4%)	12 (9.5%)	9 (7.3%)
Fractional flow reserve			
Nobs (Nmiss)	19 (231)	10 (116)	9 (115)
Median [Q1, Q3]	0.9 [0.8, 0.9]	0.9 [0.8, 0.9]	0.9 [0.9, 0.9]
Index of microvascular resistance			
Nobs (Nmiss)	14 (236)	8 (118)	6 (118)
Median [Q1, Q3]	22.0 [13.5, 26.8]	16.5 [12.8, 21.0]	28.5 [24.5, 37.0]
Resting full-cycle ratio			

	All N=250	Control N=126	Intervention N=124
Nobs (Nmiss)	7 (243)	3 (123)	4 (120)
Median [Q1, Q3]	0.9 [0.9, 0.9]	0.9 [0.9, 0.9]	0.9 [0.9, 0.9]
Coronary flow reserve			
Nobs (Nmiss)	15 (235)	9 (117)	6 (118)
Median [Q1, Q3]	3.8 [2.8, 4.9]	3.8 [3.1, 4.8]	3.4 [1.8, 4.9]

Nmiss - number missing; Q – quartile.

Table S2. Blinding effectiveness.

	All	Control	Intervention
N with pre-randomization diagnosis			
N	250	126	124
N randomised			
N	250	126	124
N with MRI diagnosis at MRI visit			
N	249	126	123
Clinician informed of randomized group allocation?			
N ($N_{MISSING}$)	245 (5)	124 (2)	121 (3)
N (%) Yes	1 (0.4%)	1 (0.8%)	0 (0.0%)
Clinician aware of randomized group allocation (if not informed)?			
N ($N_{MISSING}$)	244 (0)	123 (0)	121 (0)
N (%) Yes	0 (0.0%)	0 (0.0%)	0 (0.0%)
Participant informed of randomized group allocation?			
N ($N_{MISSING}$)	245 (5)	124 (2)	121 (3)
N (%) Yes	1 (0.4%)	1 (0.8%)	0 (0.0%)
Participant aware of randomized group allocation (if not informed)?			
N ($N_{MISSING}$)	244 (0)	123 (0)	121 (0)
N (%) Yes	0 (0.0%)	0 (0.0%)	0 (0.0%)

Table S3. Primary outcome (trial) subgroup analysis - change in diagnosis.

		Original Model	Reduced Covariate Model
Subgroup: change in diagnosis	Change in SAQ summary score: Median (Q1, Q3)	Intervention effect estimate, (95% CI), p-value	
Timepoint: 6 months			
**Diagnosis changed N=132**	**5.8 (-6.5, 30.6)**	**27.48 (21.44, 33.52), p<0.001**	**27.90 (21.92, 33.88), p<0.001**
Diagnosis unchanged N=117	5.6 (0.0, 15.2)	3.43 (-2.88, 9.75), p=0.285	3.27 (-2.99, 9.52), p=0.304
Interaction p-values:		**p<0.001**	**p<0.001**
Timepoint: 12 months			
**Diagnosis changed N=132**	**7.6 (-5.6, 33.7)**	**36.99 (30.49, 43.49), p<0.001**	**37.38 (30.95, 43.80), p<0.001**
Diagnosis unchanged N=117	5.9 (-0.9, 15.9)	4.12 (-2.67, 10.91), p=0.233	3.73 (-2.98, 10.44), p=0.275
Interaction p-value:		**p<0.001**	**p<0.001**

The model for the primary outcome analysis is extended to include terms which estimate the interaction between treatment effect and subgroups defined by a change in diagnosis between the angiography and CMR. The interaction p-value assesses if there is an interaction effect.

The original model adjusts for adjusted for baseline SAQ summary score, age, sex, diabetes, prior myocardial infarction, coronary artery disease, LV systolic function, and site (lead vs other). The reduced covariate model adjusts for the baseline SAQ summary score only. SAQ = Seattle Angina Questionnaire.

Table S4. Health-related Quality of Life Model Results.

					Original model	Reduced covariates model
Timepoint	Control<br>N=126		Intervention<br>N=124		Estimate (95% CI), p-value	Estimate (95% CI), p-value
	Follow-up value	Change from baseline	Follow-up value	Change from baseline		
Time from randomization to follow-up visit (days)						
6 month visit<br>Median [Q1, Q3]	193.0 [184.2, 207.0]		195.0 [185.0, 217.8]			
12 month visit<br>Median [Q1, Q3]	383.0 [370.0, 396.0]		386.5 [371.0, 398.0]			
EQ-5D-5L health utility index						
6 months<br>Nobs (Nmiss)	126 (0)	126 (0)	124 (0)	124 (0)		
**6 months<br>Mean (SD)**	**0.6 (0.3)**	**0.0 (0.2)**	**0.7 (0.3)**	**0.1 (0.2)**	**0.05 (0.00, 0.10), p=0.035**	**0.05 (0.01, 0.10), p=0.021**
12 months<br>Nobs (Nmiss)	126 (0)	126 (0)	124 (0)	124 (0)		
**12 months<br>Mean (SD)**	**0.6 (0.2)**	**0.0 (0.2)**	**0.7 (0.2)**	**0.1 (0.2)**	**0.09 (0.04, 0.13), p<0.001**	**0.09 (0.05, 0.14), p<0.001**
EQ-5D-5L visual analogue scale						
6 months<br>Nobs (Nmiss)	126 (0)	126 (0)	124 (0)	124 (0)		
**6 months<br>Mean (SD)**	**60.5 (18.8)**	**2.0 (15.2)**	**68.8 (16.7)**	**10.8 (17.0)**	**8.29 (4.85, 11.73), p<0.001**	**8.59 (5.17, 12.01), p<0.001**
12 months<br>Nobs (Nmiss)	126 (0)	126 (0)	124 (0)	124 (0)		
**12 months<br>Mean (SD)**	**60.4 (19.6)**	**1.8 (16.7)**	**71.6 (16.6)**	**13.7 (20.3)**	**11.13 (7.26, 15.00), p<0.001**	**11.55 (7.69, 15.40), p<0.001**

In each case, a linear regression intervention effect estimate for the 6-months and 12-months value of the score are presented. In the original model, this estimate is adjusted for the baseline value of the outcome, age, sex, diabetes, prior myocardial infarction, coronary artery disease, LV systolic function, and site (lead vs other). In the reduced covariates model, this estimate is adjusted for the baseline value of the outcome only. Nmiss = Number missing; Q = quartile; SD = standard deviation.

Table S5. Medications in the Randomised Population At Baseline and 12 months.

Medication	All N=250	Control N=126	Intervention N=124	p-value
Medication at baseline				
Any preventative therapy, n (%)	245 (98.0%)	121 (96.0%)	124 (100.0%)	p=0.060
Preventative therapy: aspirin, n (%)	213 (85.2%)	103 (81.7%)	110 (88.7%)	p=0.154
Preventative therapy: statin, n (%)	223 (89.2%)	109 (86.5%)	114 (91.9%)	p=0.221
Any angina therapy, n (%)	245 (98.0%)	123 (97.6%)	122 (98.4%)	p=1.000
Angina therapy: beta blocker, n (%)	196 (78.4%)	95 (75.4%)	101 (81.5%)	p=0.283
**Angina therapy: calcium-channel blocker, n (%)**	**78 (31.2%)**	**47 (37.3%)**	**31 (25.0%)**	**p=0.041**
Angina therapy: nitrate, n (%)	119 (47.6%)	59 (46.8%)	60 (48.4%)	p=0.899
Medication at 12 months				
**Any preventative therapy, n (%)**	**191 (76.4%)**	**85 (67.5%)**	**106 (85.5%)**	**p=0.001**
**Preventative therapy: aspirin, n (%)**	**167 (66.8%)**	**71 (56.3%)**	**96 (77.4%)**	**p<0.001**
**Preventative therapy: statin, n (%)**	**179 (71.6%)**	**78 (61.9%)**	**101 (81.5%)**	**p=0.001**
**Any angina therapy, n (%)**	**185 (74.0%)**	**81 (64.3%)**	**104 (83.9%)**	**p=0.001**
Angina therapy: beta blocker, n (%)	116 (46.4%)	59 (46.8%)	57 (46.0%)	p=0.900
**Angina therapy: calcium-channel blocker, n (%)**	**88 (35.2%)**	**34 (27.0%)**	**54 (43.5%)**	**p=0.008**
**Angina therapy: nitrate, n (%)**	**111 (44.4%)**	**41 (32.5%)**	**70 (56.5%)**	**p<0.001**

Medication use is compared between randomized groups using Fisher tests.

Table S6. Incidental findings revealed by cardiovascular magnetic resonance imaging.

Sex	Age, years	Initial diagnosis	Group (intervention or control)	Final diagnosis	True (actual) diagnosis
Female	64	Noncardiac chest pain	Intervention	Other	Myocarditis
Female	61	Noncardiac chest pain	Control	Other	Hypertrophic cardiomyopathy
Male	38	Noncardiac chest pain	Intervention	Other	Myocarditis
Male	72	Noncardiac chest pain	Control	Other	Hypertrophic cardiomyopathy

Table S7. Clinical events in the randomized population.

	Control N=126	Intervention N=124	p-value
Clinical events			
Major Adverse Cardiac and Cerebrovascular Events, n (%)	4 (3.2%)	2 (1.6%)	p=0.684
Major Adverse Cardiac Events, n (%)	4 (3.2%)	1 (0.8%)	p=0.370
All-cause mortality, n (%)	0 (0.0%)	0 (0.0%)	p=1.000
Non-fatal myocardial infarction, n (%)	1 (0.8%)	0 (0.0%)	p=1.000
Cerebrovascular event, n (%)	0 (0.0%)	1 (0.8%)	p=0.496
SAE requiring hospitalization, n (%)	11 (8.7%)	9 (7.3%)	p=0.816
Duration of serious adverse event hospital stay (days), Median [Q1, Q3]	4.0 [2.0, 5.5]	3.0 [2.0, 4.0]	p=0.611
Cardiac adverse event (hospital attendance for chest pain), n (%)	16 (12.7%)	15 (12.1%)	p=1.000
Number of cardiac adverse events (in those with at least 1), Mean (SD)	1.2 (0.6)	1.3 (0.6)	p=0.954

Values are n (%) unless otherwise specified. P-values are from the Fisher's Exact test. CAD = coronary artery disease; SD = standard deviation.

Supplementary Figure Legends

Figure S1. Scatterplots of Seattle Angina Questionnaire (SAQ) summary scores of participants in the control (n=126, blue) and intervention (n=124, red) groups at baseline and 6-months. Best-fit lines from models are fitted with the follow-up value as the outcome and baseline value as the predictor, with one model for each treatment group.

Figure S2. Scatterplots of Seattle Angina Questionnaire (SAQ) summary scores of participants in the control (n=126, blue) and intervention (n=124, red) groups at baseline and 12-months. Best-fit lines from models are fitted with the follow-up value as the outcome and baseline value as the predictor, with one model for each treatment group.

Figure S3. Scatterplots of the EuroQol-5Dimension (EQ-5D) 5-Level health-related quality of life scores of participants in the control (n=126, blue) and intervention (n=124, red) groups at baseline and 6-months. Best-fit lines from models are fitted with the follow-up value as the outcome and baseline value as the predictor, with one model for each treatment group.

Figure S4. Scatterplots of the EuroQol-5Dimension (EQ-5D) visual analog scores (VAS) of participants in the control (n=126, blue) and intervention (n=124, red) groups at baseline and 6-months. Best-fit lines from models are fitted with the follow-up value as the outcome and baseline value as the predictor, with one model for each treatment group.

Figure S5. Scatterplots of the EuroQol-5Dimension (EQ-5D) 5-Level health-related quality of life scores of participants in the control (n=126, blue) and intervention (n=124, red) groups at baseline and 12-months. Best-fit lines from models are fitted with the follow-up value as the outcome and baseline value as the predictor, with one model for each treatment group.

Figure S6. Scatterplots of the EuroQol-5Dimension (EQ-5D) visual analog scores (VAS) of participants in the control (n=126, blue) and intervention (n=124, red) groups at baseline and 12-months. Best-fit lines from

models are fitted with the follow-up value as the outcome and baseline value as the predictor, with one model for each treatment group.

Figure S7. Forest plot of standardised intervention effect estimates and 95% CIs at baseline to 6 months for the Seattle Angina Questionnaire (SAQ) and EuroQol-5Dimension (EQ-5D) (n=126 control group; n=124 intervention group). Effect estimates and corresponding confidence intervals (CI) have been standardised via dividing by the standard deviation of the change in outcome in the control population.

Figure S8. Forest plot of standardised intervention effect estimates and 95% CIs: baseline to 12 months for the Seattle Angina Questionnaire (SAQ) and EuroQol-5Dimension (EQ-5D) scores (n=126 control group; n=124 intervention group). Effect estimates and corresponding confidence intervals (CI) have been standardised via dividing by the standard deviation of the change in outcome in the control population.

Figure S9. A 61-year-old woman with a history of chronic chest pain was referred from general practice for an outpatient cardiology assessment. A treadmill exercise test using the Bruce protocol (A) was completed with no ST-segment changes; the exercise duration was 9:01 minutes, the stopping reason was fatigue and the patient remained asymptomatic during and after the exercise test. Due to ongoing chest pain despite two anti-anginal medications the patient was referred for an invasive coronary angiogram. The angiogram subsequently revealed unobstructed coronary arteries without evidence of atherosclerosis (B).

Following the angiogram, the patient was given an information document for the CorCMR study (ClinicalTrials.gov NCT04805814) and written informed consent was subsequently provided. Intravenous adenosine (140 - 210 µg/kg/min) stress/rest cardiovascular magnetic resonance (CMR) myocardial perfusion imaging was undertaken on a 1.5 Tesla scanner (MAGNETOM Avanto Fit, Siemens Healthcare) within three months of the angiogram. Cine imaging (C) revealed preserved left ventricular systolic function and normal cardiac dimensions. The myocardial native longitudinal relaxation (T1) time (D, 998ms) and native transverse relaxation (T2) time (E, 47ms) were normal, and there was no evidence of late gadolinium enhancement on contrast-enhanced imaging (0.2 mmol/kg (total dose), Gadovist®, Bayer Healthcare) (F). Adenosine stress (first-pass contrast, 0.05 mmol/kg Gadovist®) and rest (first-pass contrast, 0.05 mmol/kg Gadovist®) dynamic

CMR imaging (G) coupled with inline pixel mapping of myocardial blood flow revealed normal myocardial perfusion. The global myocardial blood flow during first-pass adenosine stress imaging was 3.98ml/min/g (normal reference range ≥2.25ml/min/g) and the global myocardial perfusion reserve was 4.10 (reference range ≥2.2). The final diagnosis was non-cardiac chest pain.

Figure S10. A 70-year-old woman with a history of recurrent hospitalizations for chest pain was referred from primary care for assessment at the cardiology outpatient clinic. The hospital visits had been consistently associated with troponin I concentrations that were within the normal range (<16 ng/L sex-specific 99th centile for high-sensitivity troponin, ARCHITECT Abbott). A Bruce protocol treadmill exercise test was undertaken at the cardiology clinic. The patient experienced chest discomfort during the test and the exercise electrocardiogram revealed multi-territory horizontal ST-segment depression (maximum 3.5 mm, lead V5) occurring during Stage II (3:05 minutes). These features were indicative of inducible myocardial ischemia (A) and the patient was referred for an invasive coronary angiogram. The angiogram subsequently revealed multivessel, diffuse, nonobstructive atherosclerotic plaque (B).

The patient was then given an Information Sheet for the CorCMR study (NCT04805814) and written informed consent was subsequently provided. An intravenous adenosine (140 µg/kg/min) stress/rest CMR scan (MAGNETOM Avanto Fit, Siemens Healthcare) was undertaken as part of the study protocol. Cine imaging (C) revealed preserved left ventricular systolic function and normal cardiac dimensions. Native myocardial T1 (D, 1002ms) and T2 (E, 46ms) relaxation times were normal. There was no evidence of late gadolinium enhancement on contrast-enhanced CMR imaging (0.20 mmol/kg (total dose) Gadovist®) (F). C- Adenosine stress / rest dynamic CMR imaging of first-pass bolus of contrast media (0.05 mmol/kg Gadovist®) coupled with inline pixel mapping of myocardial blood flow revealed a circumferential subendocardial perfusion defect, an abnormally low global hyperaemic myocardial blood flow (1.80ml/min/g), and a reduced myocardial perfusion reserve (1.67). The final diagnosis was microvascular angina.

Supplementary Figures

Figure S1.

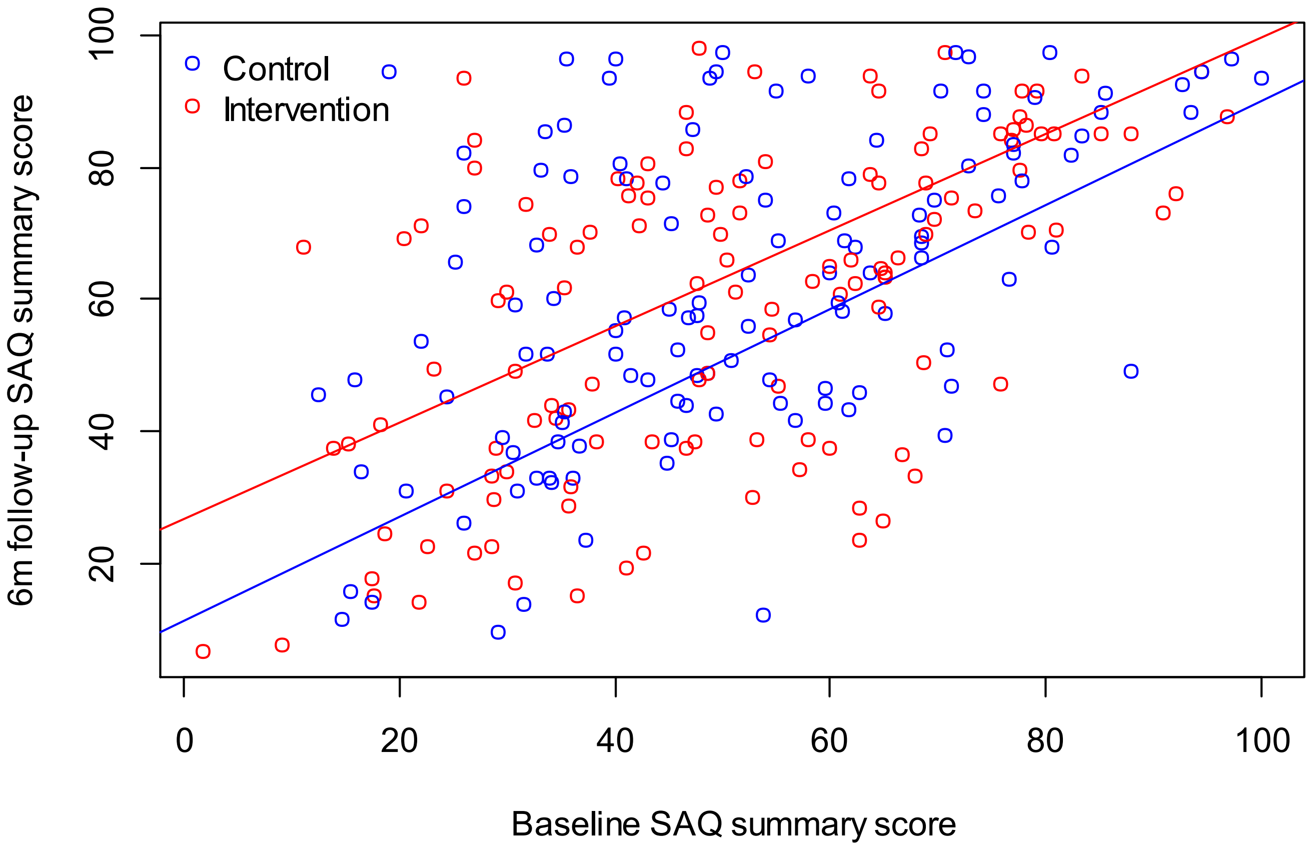

Figure S2.

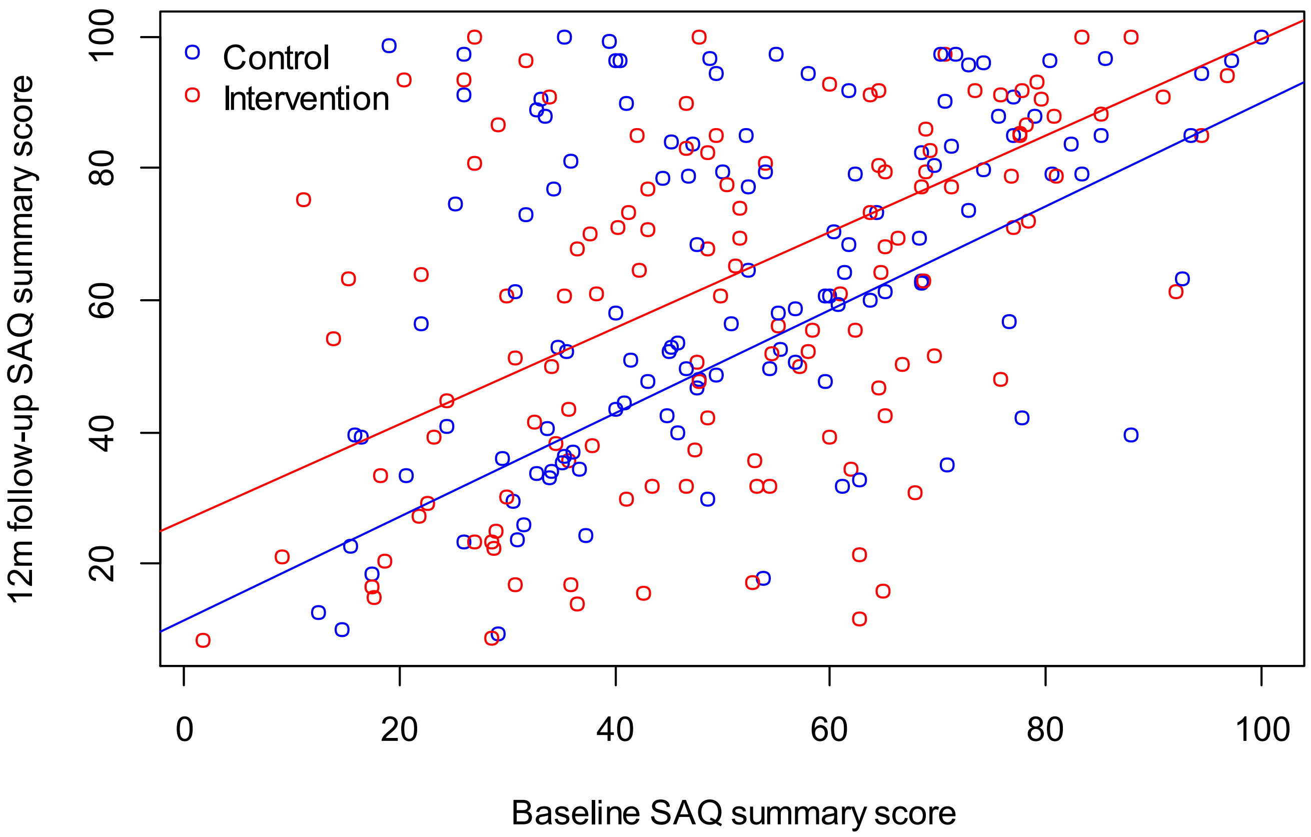
Control
Intervention
12m follow-up SAQ summary score
Baseline SAQ summary score
0
20
40
60
80
100
20
40
60
80
100

Figure S3.

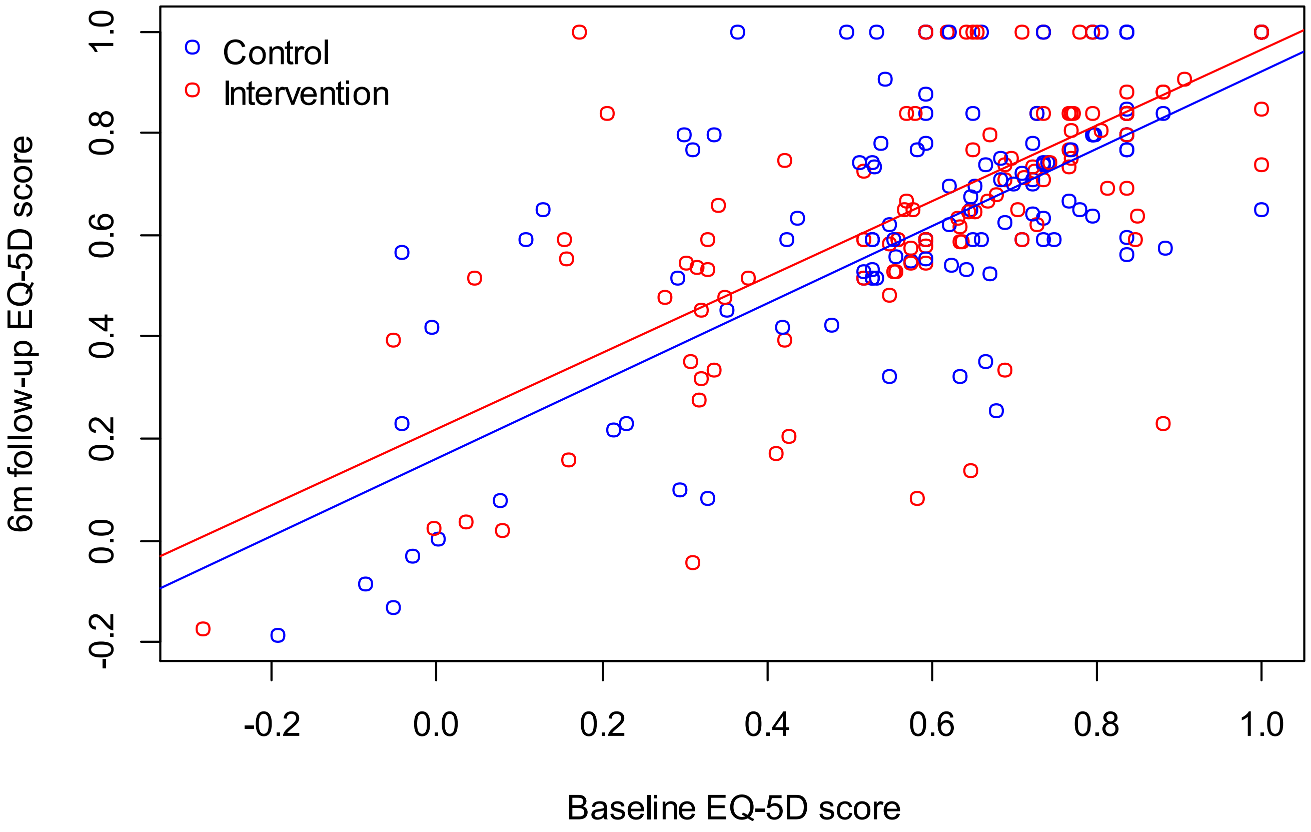

Control
Intervention
6m follow-up EQ-5D score
-0.2
0.0
0.2
0.4
0.6
0.8
1.0
-0.2
0.0
0.2
0.4
0.6
0.8
1.0
Baseline EQ-5D score

Figure S4.

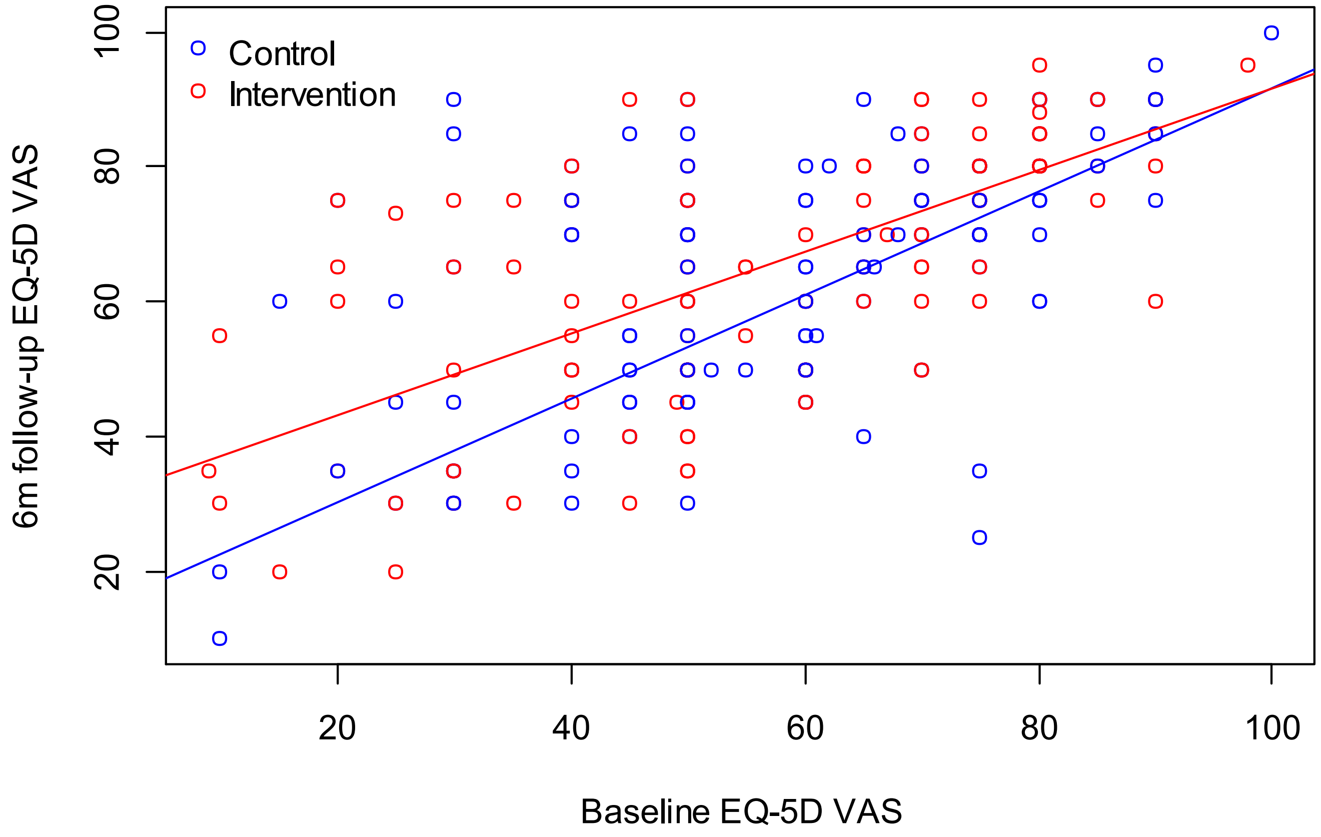
Control
Intervention
6m follow-up EQ-5D VAS
Baseline EQ-5D VAS
20
40
60
80
100
20
40
60
80
100

Figure S5.

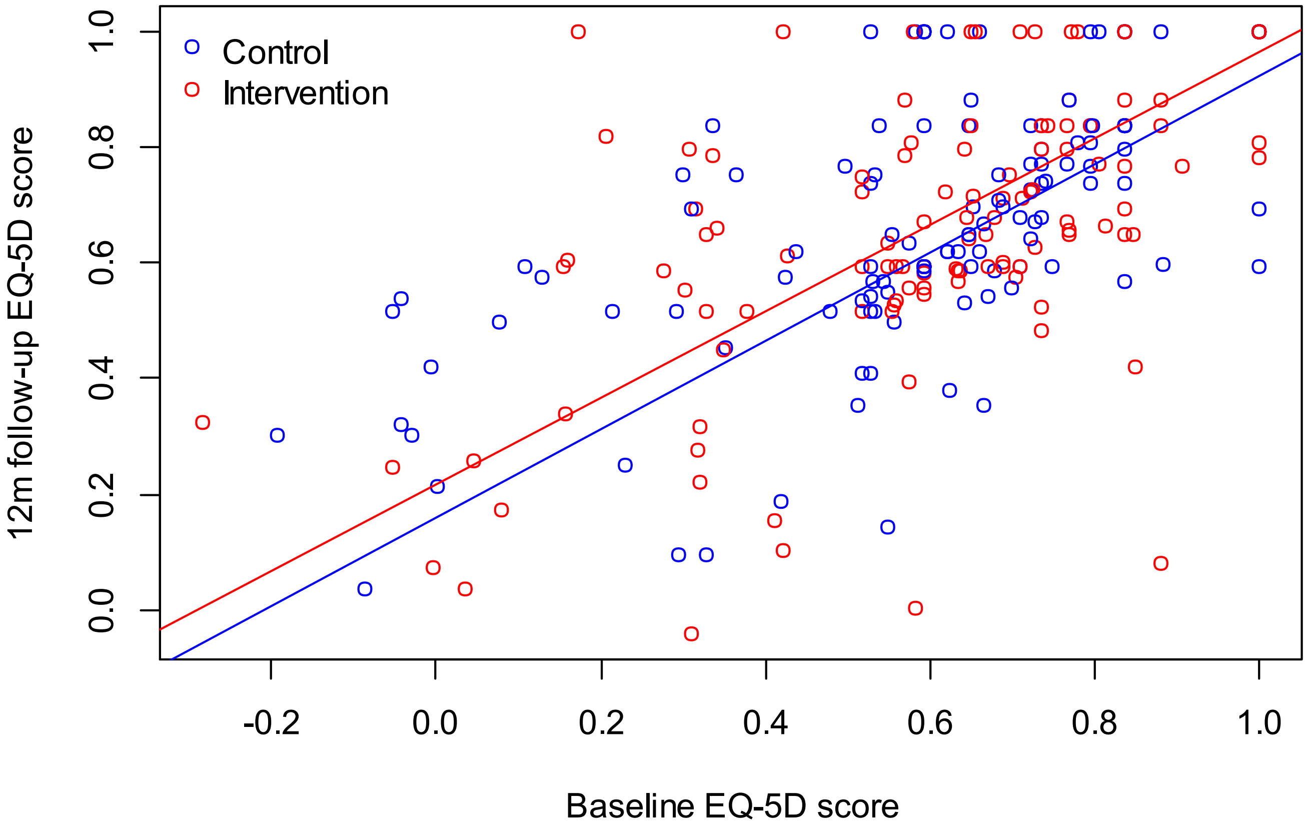
Control
Intervention
12m follow-up EQ-5D score
Baseline EQ-5D score
-0.2
0.0
0.2
0.4
0.6
0.8
1.0
0.0
0.2
0.4
0.6
0.8
1.0

Figure S6.

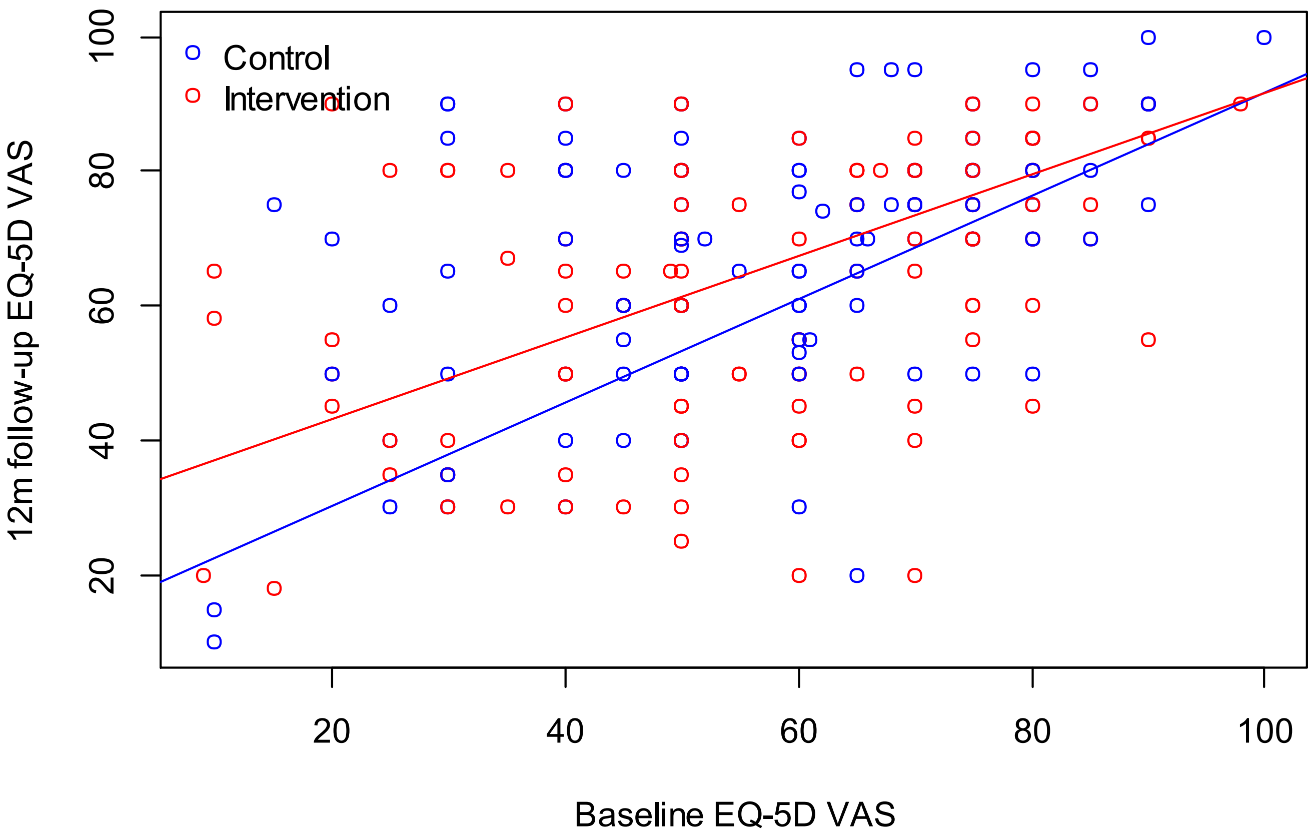
Control
Intervention
12m follow-up EQ-5D VAS
20
40
60
80
100
20
40
60
80
100
Baseline EQ-5D VAS

Figure S7.

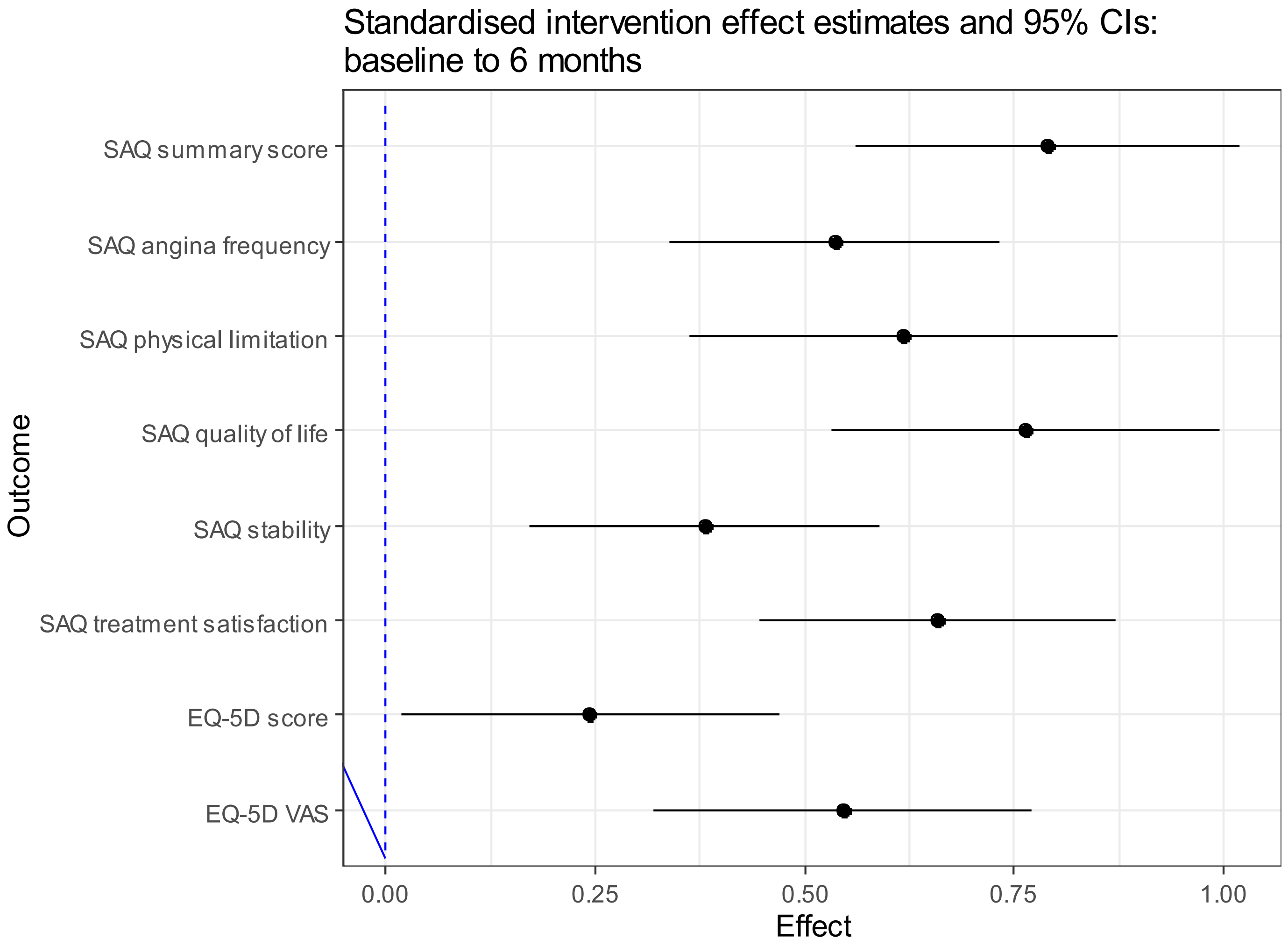

Standardised intervention effect estimates and 95% CIs:
baseline to 6 months
SAQ summary score
SAQ angina frequency
SAQ physical limitation
SAQ quality of life
SAQ stability
SAQ treatment satisfaction
EQ-5D score
EQ-5D VAS
Outcome
0.00
0.25
0.50
0.75
1.00
Effect

Figure S8.

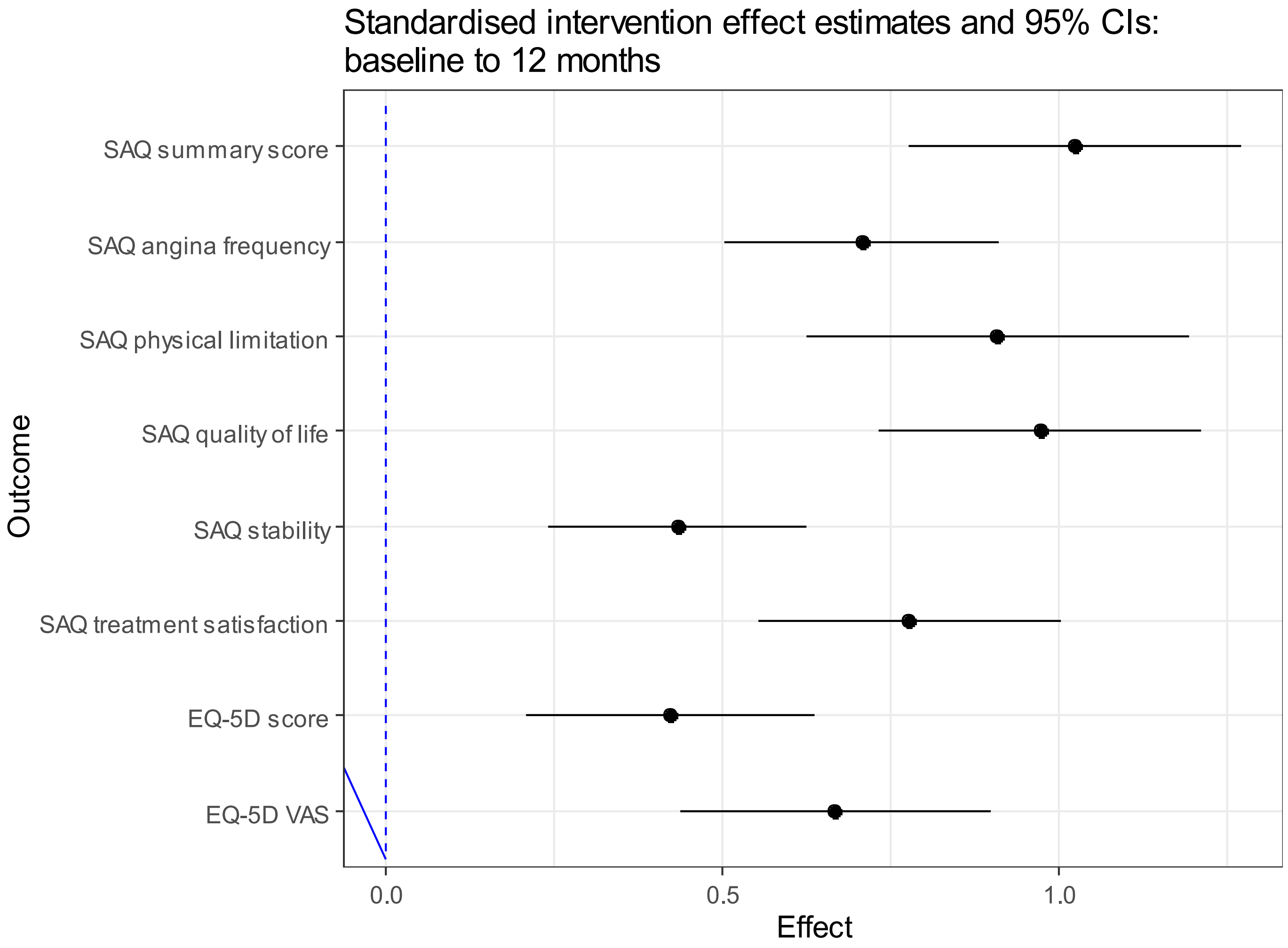
Standardised intervention effect estimates and 95% CIs:
baseline to 12 months
SAQ summary score
SAQ angina frequency
SAQ physical limitation
SAQ quality of life
SAQ stability
SAQ treatment satisfaction
EQ-5D score
EQ-5D VAS
Outcome
0.0
0.5
1.0
Effect

Figure S9.

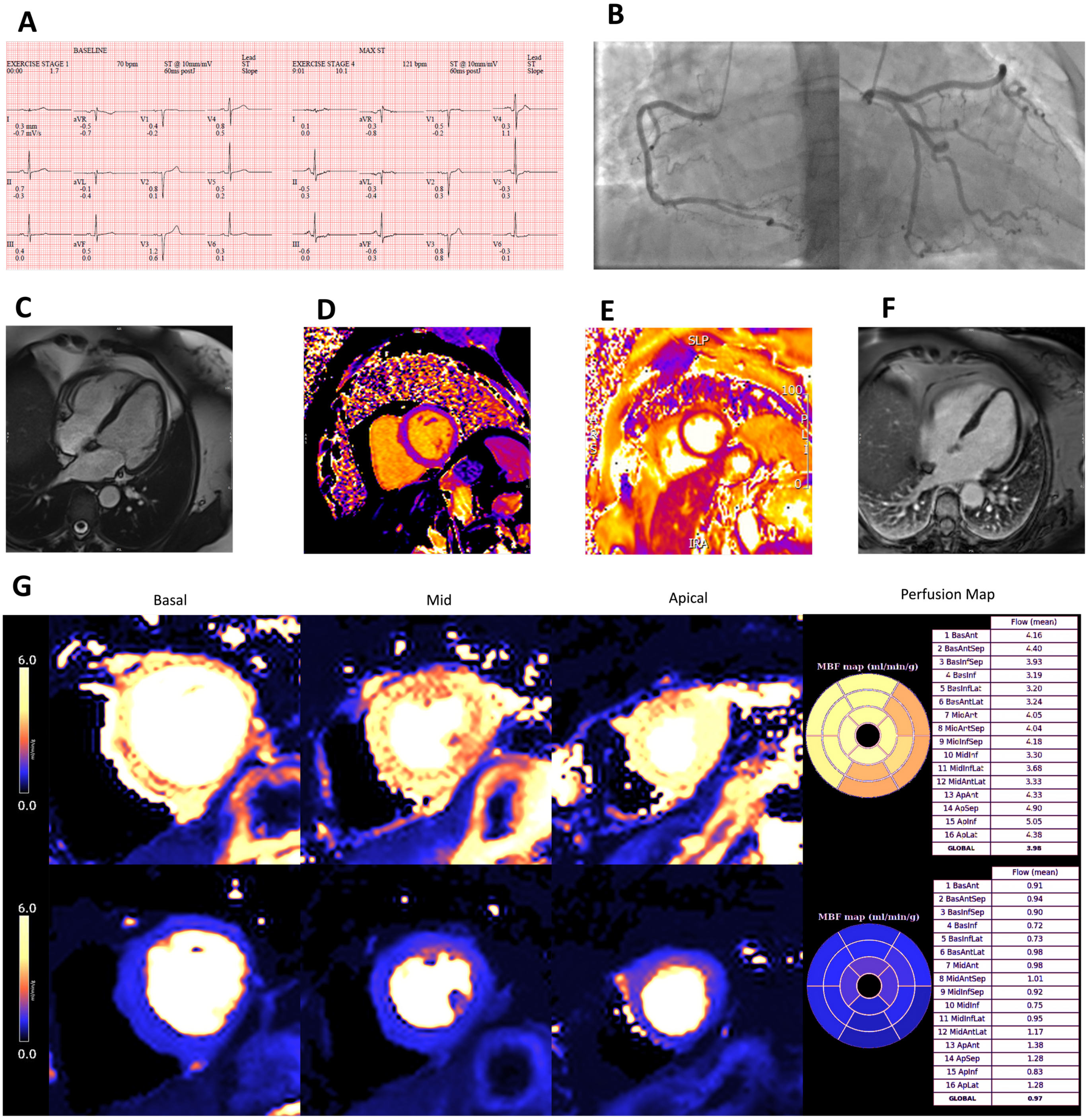
A
B
C
D
E
F
G
BASELINE
MAX ST
SLP
IRA
Basal
Mid
Apical
Perfusion Map
Stress
Rest
6.0
0.0
MBF map (ml/min/g)
Flow (mean)
1 BasAnt 4.16
2 BasAntSep 4.40
3 BasInfSep 3.93
4 BasInf 3.19
5 BasInfLat 3.20
6 BasAntLat 3.24
7 MidAnt 4.05
8 MidAntSep 4.04
9 MidInfSep 4.18
10 MidInf 3.30
11 MidInfLat 3.68
12 MidAntLat 3.33
13 ApAnt 4.33
14 ApSep 4.90
15 ApInf 5.05
16 ApLat 4.38
GLOBAL 3.98
MBF map (ml/min/g)
Flow (mean)
1 BasAnt 0.91
2 BasAntSep 0.94
3 BasInfSep 0.90
4 BasInf 0.72
5 BasInfLat 0.73
6 BasAntLat 0.98
7 MidAnt 0.98
8 MidAntSep 1.01
9 MidInfSep 0.92
10 MidInf 0.75
11 MidInfLat 0.95
12 MidAntLat 1.17
13 ApAnt 1.38
14 ApSep 1.28
15 ApInf 0.83
16 ApLat 1.28
GLOBAL 0.97

Figure S10.

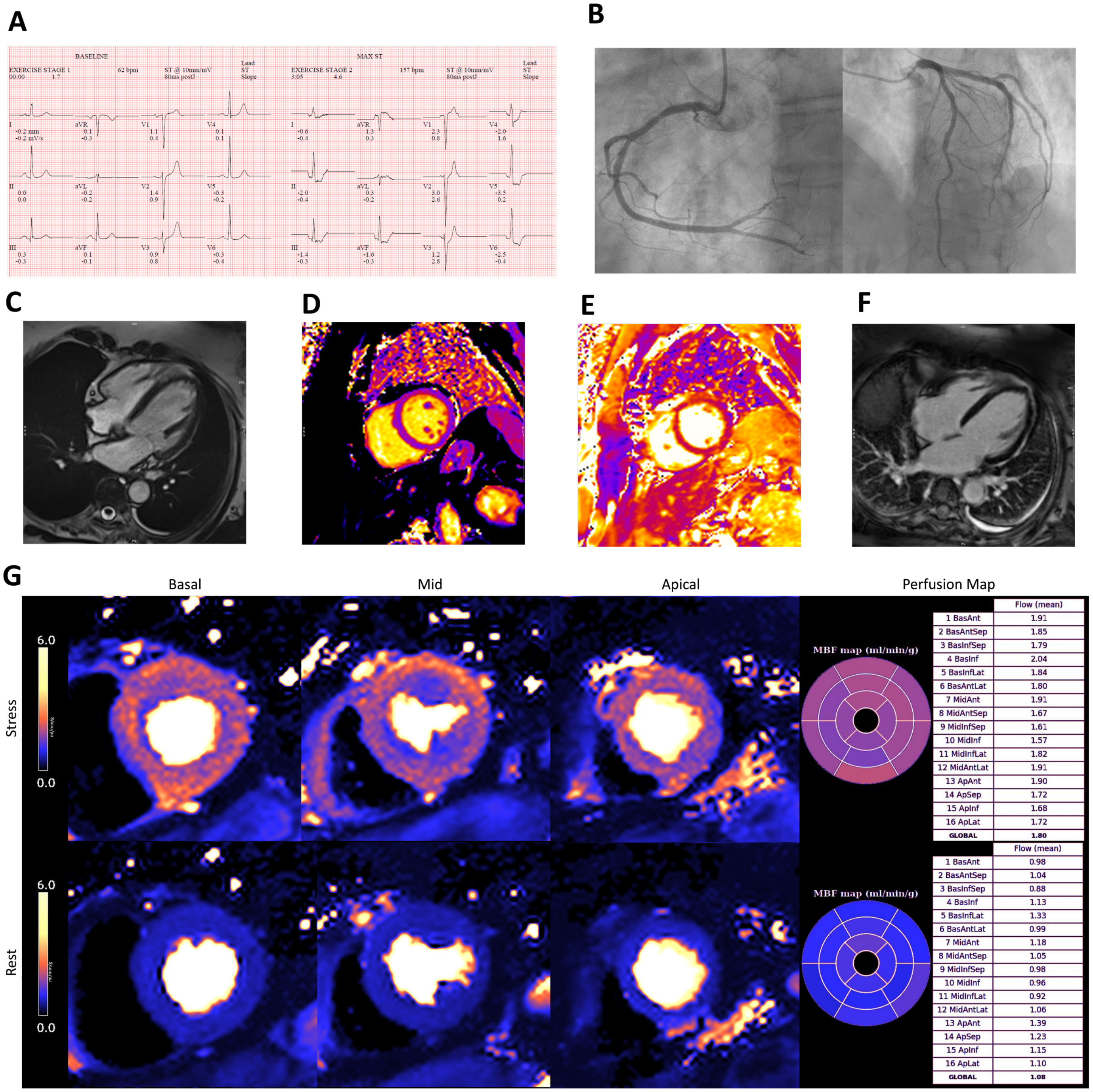
A
B
C
D
E
F
G
BASELINE
MAX ST
Basal
Mid
Apical
Perfusion Map
Stress
Rest
6.0
0.0
MBF map (ml/min/g)
Flow (mean)
1 BasAnt 1.91
2 BasAntSep 1.85
3 BasInfSep 1.79
4 BasInf 2.04
5 BasInfLat 1.84
6 BasAntLat 1.80
7 MidAnt 1.91
8 MidAntSep 1.67
9 MidInfSep 1.61
10 MidInf 1.57
11 MidInfLat 1.82
12 MidAntLat 1.91
13 ApAnt 1.90
14 ApSep 1.72
15 ApInf 1.68
16 ApLat 1.72
GLOBAL 1.80
MBF map (ml/min/g)
Flow (mean)
1 BasAnt 0.98
2 BasAntSep 1.04
3 BasInfSep 0.88
4 BasInf 1.13
5 BasInfLat 1.33
6 BasAntLat 0.99
7 MidAnt 1.18
8 MidAntSep 1.05
9 MidInfSep 0.98
10 MidInf 0.96
11 MidInfLat 0.92
12 MidAntLat 1.06
13 ApAnt 1.39
14 ApSep 1.23
15 ApInf 1.15
16 ApLat 1.10
GLOBAL 1.08

Clinician guidance letter by diagnosis

The information is based on guidelines from the European Society of Cardiology [15]. The guidance is intended for the attending cardiologist and general practitioner. The guidance will be provided as an accompanying discharge letter following the invasive coronary angiogram.

<insert hospital letterhead>

TITLE: The Clinical Utility Of Cardiac Magnetic Resonance Imaging in Patients With Angina But No Obstructive Coronary Disease (CorCMR): A Diagnostic Study And Nested Randomised Trial.

– Clinician guidance and recommended therapy

Patient details: insert label

Diagnosis – Microvascular angina

We have provided brief guidance to assist in managing microvascular angina based on the guidelines from the European Society of Cardiology (2019), SIGN (2017) and British Heart Foundation.[1-3]

Pharmacological management

- Calcium antagonists (e.g. **Verapamil** 40mg BD up-titrated weekly according to response); NB do not combine a rate limiting calcium channel blocker (verapamil, diltiazem) with a beta blocker.
    - Or Beta-blockers (e.g. 1.25mg **Bisoprolol** up-titrated or alternatively 3.125 mg of carvedilol twice daily with up-titration if feasible and appropriate, see Summary of Product Characteristics; https://www.medicines.org.uk/emc/medicine/27714)
- **Aspirin, Statin** or **ACEI** may be reasonable (depending on patient characteristics)
- Short-acting PRN nitrate (e.g. **Sublingual GTN**)
- **Nicorandil** if refractory symptoms (e.g. 5mg BD up-titrated weekly according to response)
- Xanthine inhibitors (aminophylline) – if refractory to all above

Non-Pharmacological lifestyle & risk factor control

- **Smoking** "Smoking is a strong and independent risk factor for CVD and all smoking, including environmental smoking exposure, must be avoided in all patients with CVD"
- **Diet** "A healthy diet reduces CVD risk... Energy intake should be limited to the amount of energy needed to maintain (or obtain) a healthy weight—that is, a BMI <25 kg/m2."
- **Exercise** "moderate-to-vigorous intensity aerobic exercise training ⩾3 times a week" (30 min)
- **Weight** "Weight reduction in overweight and obese people is recommended in order to achieve favourable effects on BP, dyslipidaemia and glucose metabolism"
- **Lipids –** "The goals of treatment are LDL-C below 1.8 mmol/L"
- **Hypertension –** "Blood pressure to values within the range 130–139/80–85 mmHg"
- **Diabetes** "good control of glycated haemoglobin (HbA1c) to <7.0%...based on individual considerations."
- **Psychosocial** "Patients should be assessed for psychosocial distress and appropriate care offered… Refer for psychotherapy, medication or collaborative care in the case of clinically significant symptoms of depression, anxiety and hostility."
- **Cardiac rehabilitation** "A comprehensive risk-reduction regimen, integrated into comprehensive cardiac rehabilitation, is recommended."

References

1. Knuuti J, Wijns W, Saraste A, Capodanno D, Barbato E, Funck-Brentano C, Prescott E, Storey RF, Deaton C, Cuisset T, Agewall S, Dickstein K, Edvardsen T, Escaned J, Gersh BJ, Svitil P, Gilard M, Hasdai D, Hatala R, Mahfoud F, Masip J, Muneretto C, Valgimigli M, Achenbach S, Bax JJ; ESC Scientific Document Group. 2019 ESC Guidelines for the diagnosis and management of chronic coronary syndromes. Eur Heart J. 2019 Aug 31. pii: ehz425.
2. SIGN. Guideline No. 96 - Management of stable angina. Edinburgh: Scottish Intercollegiate Guidelines Network (SIGN); 2007.
3. British Heart Foundation. https://www.bhf.org.uk/informationsupport/conditions/angina

Clinician guidance letter by diagnosis

<insert GJNH letterhead>

TITLE: The Clinical Utility Of Cardiac Magnetic Resonance Imaging in Patients With Angina But No Obstructive Coronary Disease (CorCMR): A Diagnostic Study And Nested Randomised Trial.

– Clinician guidance and recommended therapy

Patient details: insert label

Diagnosis – Vasospastic angina

We have provided brief guidance to assist in managing vasospastic angina based on the 2019 ESC guidelines & 2007 SIGN guidelines.[1,2]

Pharmacological management

- Non-dihydropyridine calcium channel blocker (e.g. **Verapamil** initially 40mg BD increasing at weekly intervals as tolerated up to 240-360 mg daily)
- +/- **Long-acting nitrates** if symptoms ongoing (scheduled to cover the period of the day in which ischaemic episodes most frequently occur, in order to prevent nitrate tolerance.
- β-Blockers should be avoided.
- **Aspirin, statin and ACE-I** therapy may be reasonable, and is recommended if coronary disease is revealed by coronary angiography (CTCA or invasive)

Non-Pharmacological lifestyle & risk factor control

- **Specific to vasospastic angina –** "exclude cocaine/amphetamine use"
- **Smoking** "Smoking is a strong and independent risk factor for CVD and all smoking, including environmental smoking exposure, must be avoided in all patients with CVD"
- **Diet** "A healthy diet reduces CVD risk... Energy intake should be limited to the amount of energy needed to maintain (or obtain) a healthy weight—that is, a BMI <25 kg/m2."
- **Exercise** "moderate-to-vigorous intensity aerobic exercise training ⩾3 times a week" (30 min)
- **Weight** "Weight reduction in overweight and obese people is recommended in order to achieve favourable effects on BP, dyslipidaemia and glucose metabolism"
- **Lipids –** "The goals of treatment are LDL-C below 1.8 mmol/L"

- **Hypertension** – “SBP/DBP to values within the range 130–139/80–85 mmHg”
- **Diabetes** “good control of glycated haemoglobin (HbA1c) to <7.0%...based on individual considerations.”
- **Psychosocial** “Patients should be assessed for psychosocial distress and appropriate care offered… Refer for psychotherapy, medication or collaborative care in the case of clinically significant symptoms of depression, anxiety and hostility.”

Cardiac rehabilitation “A comprehensive risk-reduction regimen, integrated into comprehensive cardiac rehabilitation, is recommended.

References

1. Knuuti J, Wijns W, Saraste A, Capodanno D, Barbato E, Funck-Brentano C, Prescott E, Storey RF, Deaton C, Cuisset T, Agewall S, Dickstein K, Edvardsen T, Escaned J, Gersh BJ, Svitil P, Gilard M, Hasdai D, Hatala R, Mahfoud F, Masip J, Muneretto C, Valgimigli M, Achenbach S, Bax JJ; ESC Scientific Document Group. 2019 ESC Guidelines for the diagnosis and management of chronic coronary syndromes. Eur Heart J. 2019 Aug 31. pii: ehz425.
2. SIGN. Guideline No. 96 - Management of stable angina. Edinburgh: Scottish Intercollegiate Guidelines Network (SIGN); 2007.
3. British Heart Foundation. https://www.bhf.org.uk/informationsupport/conditions/angina

Patient guidance letter by diagnosis

– Clinician guidance and recommended therapy

Patient details: insert label

Diagnosis – Obstructive (flow-limiting) coronary artery disease

We have provided brief guidance to assist the care of patients with flow-limiting (obstructive) coronary artery disease based on the guidelines from the European Society of Cardiology (2019), SIGN (2017) and British Heart Foundation.[1-3]

Pharmacological management

- Angina medication
  - Beta-blockers (e.g. 2.5mg **Bisoprolol** uptitrated or 3.125 mg of carvedilol twice daily with up-titration if feasible and appropriate, see Summary of Product Characteristics; https://www.medicines.org.uk/emc/medicine/27714)
  - Isosorbide mononitrate
  - Calcium antagonist e.g. **Verapamil** 40mg BD uptitrated weekly according to response; NB do not combine a rate limiting calcium channel blocker (verapamil, diltiazem) with a beta blocker.
- **Aspirin, Statin** or **ACEI** may be reasonable (depending on patient characteristics)
- Short-acting PRN nitrate (e.g. **Sublingual GTN**)
- **Nicorandil** if refractory symptoms (e.g. 5mg BD uptitrated weekly according to response)

Non-Pharmacological lifestyle & risk factor control

- **Smoking** "Smoking is a strong and independent risk factor for CVD and all smoking, including environmental smoking exposure, must be avoided in all patients with CVD"
- **Diet** "A healthy diet reduces CVD risk... Energy intake should be limited to the amount of energy needed to maintain (or obtain) a healthy weight—that is, a BMI <25 kg/m2."
- **Exercise** "moderate-to-vigorous intensity aerobic exercise training ⩾3 times a week" (30 min)
- **Weight** "Weight reduction in overweight and obese people is recommended in order to achieve favourable effects on BP, dyslipidaemia and glucose metabolism"
- **Lipids** – "The goals of treatment are LDL-C below 1.8 mmol/L"
- **Hypertension** – "Blood pressure to values within the range 130–139/80–85 mmHg"

- **Diabetes** “good control of glycated haemoglobin (HbA1c) to <7.0%...based on individual considerations.”
- **Psychosocial** “Patients should be assessed for psychosocial distress and appropriate care offered… Refer for psychotherapy, medication or collaborative care in the case of clinically significant symptoms of depression, anxiety and hostility.”
- **Cardiac rehabilitation** “A comprehensive risk-reduction regimen, integrated into comprehensive cardiac rehabilitation, is recommended.”

References

1. Knuuti J, Wijns W, Saraste A, Capodanno D, Barbato E, Funck-Brentano C, Prescott E, Storey RF, Deaton C, Cuisset T, Agewall S, Dickstein K, Edvardsen T, Escaned J, Gersh BJ, Svitil P, Gilard M, Hasdai D, Hatala R, Mahfoud F, Masip J, Muneretto C, Valgimigli M, Achenbach S, Bax JJ; ESC Scientific Document Group. 2019 ESC Guidelines for the diagnosis and management of chronic coronary syndromes. Eur Heart J. 2019 Aug 31. pii: ehz425.
2. SIGN. Guideline No. 96 - Management of stable angina. Edinburgh: Scottish Intercollegiate Guidelines Network (SIGN); 2007.
3. British Heart Foundation. https://www.bhf.org.uk/informationsupport/conditions/angina

Patient guidance letter by diagnosis

– Clinician guidance and recommended therapy

Patient details: insert label

Diagnosis – No clinically significant coronary disease

A coronary angiogram was recently undertaken where no obstructive coronary disease was identified. The findings support a diagnosis of non-cardiac chest pain.

Pharmacological management

- Anginal medication may not be needed and may be discontinued.

Non-Pharmacological lifestyle & risk factor control

The following advice should be considered for general wellbeing.

- **Smoking** "Smoking is a strong and independent risk factor for CVD and all smoking, including environmental smoking exposure, must be avoided in all patients with CVD"
- **Diet** "A healthy diet reduces CVD risk... Energy intake should be limited to the amount of energy needed to maintain (or obtain) a healthy weight—that is, a BMI <25 kg/m2."
- **Exercise** "moderate-to-vigorous intensity aerobic exercise training ⩾3 times a week" (30 min)
- **Weight** "Weight reduction in overweight and obese people is recommended in order to achieve favourable effects on BP, dyslipidaemia and glucose metabolism"
- **Lipids** – "The goals of treatment are LDL-C below 1.8 mmol/L"
- **Hypertension** – "Blood pressure to values within the range 130–139/80–85 mmHg"
- **Diabetes** "good control of glycated haemoglobin (HbA1c) to <7.0%...based on individual considerations."
- **Psychosocial** "Patients should be assessed for psychosocial distress and appropriate care offered… Refer for psychotherapy, medication or collaborative care in the case of clinically significant symptoms of depression, anxiety and hostility."
- **Cardiac rehabilitation** "A comprehensive risk-reduction regimen, integrated into comprehensive cardiac rehabilitation, is recommended."

References

1. Knuuti J, Wijns W, Saraste A, Capodanno D, Barbato E, Funck-Brentano C, Prescott E,

Storey RF, Deaton C, Cuisset T, Agewall S, Dickstein K, Edvardsen T, Escaned J, Gersh BJ, Svitil P, Gilard M, Hasdai D, Hatala R, Mahfoud F, Masip J, Muneretto C, Valgimigli M, Achenbach S, Bax JJ; ESC Scientific Document Group. 2019 ESC Guidelines for the diagnosis and management of chronic coronary syndromes. Eur Heart J. 2019 Aug 31. pii: ehz425.

Patient guidance letter by diagnosis

This guidance is intended for all patients with management advice provided in line with the endotype (diagnosis). The information is based on guidelines from the European Society of Cardiology and SIGN. The guidance is intended for the patient. The guidance will be provided following the invasive coronary angiogram.

<insert hospital letterhead>

CorCMR Study – patient guideline

Diagnosis – Microvascular angina

Medication
Taking your medication is important to control your symptoms. If you feel that your symptoms are not adequately controlled, please see your GP to have them adjusted.

Smoking
All smoking, including environmental smoking exposure, must be avoided. Smoking is a strong and independent risk factor for heart disease.
For more information, visit:
https://www.bhf.org.uk/heart-health/risk-factors/smoking

Diet
A healthy diet reduces the risk of heart disease. Calorie intake should be limited to the amount of energy needed to maintain (or obtain) a healthy weight—that is, a BMI of <25 kg/m^2.
For more information, visit:
https://www.bhf.org.uk/heart-health/preventing-heart-disease/healthy-eating

Exercise
Keeping active is important. You should exercise for 30 minutes ≥3 times a week.
For more information, visit:
https://www.bhf.org.uk/heart-health/preventing-heart-disease/staying-active

Weight
Weight reduction (if overweight) is recommended in order to achieve favourable effects on blood pressure, cholesterol, and diabetes control.
For more information, visit:
https://www.bhf.org.uk/heart-health/preventing-heart-disease/managing-your-weight

Cholesterol
Aim for a normal cholesterol level. Please have your GP check your cholesterol and modify your medication until this is achieved.
For more information, visit:
https://www.bhf.org.uk/heart-health/risk-factors/high-cholesterol

High blood pressure
Your blood pressure should be within the range of 130–139/80–85 mmHg – please have your GP check your blood pressure and modify your medication until this is achieved.
For more information, visit:
https://www.bhf.org.uk/heart-health/risk-factors/high-blood-pressure

Diabetes
A healthy, low-sugar diet and regular exercise will help.
For more information, visit:
https://www.bhf.org.uk/heart-health/risk-factors/diabetes

Stress
If you suffer from anxiety or depression, psychotherapy, medication and collaborative care are available through the primary care service and can be offered to you by your GP.
For more information, visit
https://www.bhf.org.uk/heart-health/preventing-heart-disease/stress
https://www.bhf.org.uk/heart-health/preventing-heart-disease/heart-and-mental-health

Cardiac rehabilitation
A cardiac rehabilitation programme is recommended to all patients with heart disease. You have been referred to your local cardiac rehabilitation service – please attend for your appointment.

Patient guidance letter by diagnosis

<insert hospital letterhead>

CorCMR Study – patient guideline

Diagnosis – Vasospastic angina

Medication
Taking your medication is important to control your symptoms. If you feel that your symptoms are not adequately controlled, please see your GP to have them adjusted.

Smoking
All smoking, including environmental smoking exposure, must be avoided. Smoking is a strong and independent risk factor for heart disease.
For more information, visit:
https://www.bhf.org.uk/heart-health/risk-factors/smoking

Diet
A healthy diet reduces the risk of heart disease. Calorie intake should be limited to the amount of energy needed to maintain (or obtain) a healthy weight—that is, a BMI of <25 kg/m^2.
For more information, visit:
https://www.bhf.org.uk/heart-health/preventing-heart-disease/healthy-eating

Exercise
Keeping active is important. You should exercise for 30 minutes ≥3 times a week.
For more information, visit:
https://www.bhf.org.uk/heart-health/preventing-heart-disease/staying-active

Weight
Weight reduction (if overweight) is recommended in order to achieve favourable effects on blood pressure, cholesterol, and diabetes control.
For more information, visit:
https://www.bhf.org.uk/heart-health/preventing-heart-disease/managing-your-weight

Cholesterol
Aim for a normal cholesterol level. Please have your GP check your cholesterol and modify your medication until this is achieved.
For more information, visit:
https://www.bhf.org.uk/heart-health/risk-factors/high-cholesterol

High blood pressure
Your blood pressure should be within the range of 130–139/80–85 mmHg – please have your GP check your blood pressure and modify your medication until this is achieved.
For more information, visit:
https://www.bhf.org.uk/heart-health/risk-factors/high-blood-pressure

Diabetes
A healthy, low-sugar diet and regular exercise will help.
For more information, visit:
https://www.bhf.org.uk/heart-health/risk-factors/diabetes

Stress
If you suffer from anxiety or depression, psychotherapy, medication and collaborative care are available through the primary care service and can be offered to you by your GP.
For more information, visit
https://www.bhf.org.uk/heart-health/preventing-heart-disease/stress
https://www.bhf.org.uk/heart-health/preventing-heart-disease/heart-and-mental-health

Cardiac rehabilitation
A cardiac rehabilitation programme is recommended to all patients with heart disease. You have been referred to your local cardiac rehabilitation service – please attend for your appointment.

Patient guidance letter by diagnosis

<insert hospital letterhead>

CorCMR Study – patient guideline

Diagnosis – Obstructive Coronary Artery Disease

Medication
Your prescribed medications are important to reduce the risk of heart attacks and to control your symptoms. If you feel that your symptoms are not adequately controlled, please see your GP to have them adjusted and/or re-referral to the cardiology service. If you have on-going anginal symptoms despite medication then a stent or bypass surgery may be helpful.

Smoking
All smoking, including environmental smoking exposure, must be avoided. Smoking is a strong and independent risk factor for heart disease.
For more information, visit:
https://www.bhf.org.uk/heart-health/risk-factors/smoking

Diet
A healthy diet reduces the risk of heart disease. Calorie intake should be limited to the amount of energy needed to maintain (or obtain) a healthy weight—that is, a BMI of <25 kg/m^2.
For more information, visit:
https://www.bhf.org.uk/heart-health/preventing-heart-disease/healthy-eating

Exercise
Keeping active is important. You should exercise for 30 minutes ≥3 times a week.
For more information, visit:
https://www.bhf.org.uk/heart-health/preventing-heart-disease/staying-active

Weight
Weight reduction (if overweight) is recommended in order to achieve favourable effects on blood pressure, cholesterol, and diabetes control.
For more information, visit:
https://www.bhf.org.uk/heart-health/preventing-heart-disease/managing-your-weight

Cholesterol
Aim for a normal cholesterol level. Please have your GP check your cholesterol and modify your medication until this is achieved.
For more information, visit:
https://www.bhf.org.uk/heart-health/risk-factors/high-cholesterol

High blood pressure
Your blood pressure should be within the range of 130–139/80–85 mmHg – please have your GP check your blood pressure and modify your medication until this is achieved.
For more information, visit:

https://www.bhf.org.uk/heart-health/risk-factors/high-blood-pressure

Diabetes
A healthy, low-sugar diet and regular exercise will help.
For more information, visit:
https://www.bhf.org.uk/heart-health/risk-factors/diabetes

Stress
If you suffer from anxiety or depression, psychotherapy, medication and collaborative care are available through the primary care service and can be offered to you by your GP.
For more information, visit
https://www.bhf.org.uk/heart-health/preventing-heart-disease/stress
https://www.bhf.org.uk/heart-health/preventing-heart-disease/heart-and-mental-health

Cardiac rehabilitation
A cardiac rehabilitation programme is recommended to all patients with heart disease. You have been referred to your local cardiac rehabilitation service – please attend for your appointment.

Patient guidance letter by diagnosis

<insert hospital letterhead>

CorCMR Study – patient guideline

Diagnosis – Non-cardiac chest pain

Symptom management
We have excluded heart disease as a cause of your chest discomfort. This is reassuring.
If further tests or referrals have been organised for you, please attend for your appointment.
If your symptoms persist, please see your GP.

Medication
Some or all of your heart medication can now be reduced or stopped.
Please discuss this with your GP.

Prevention of future heart disease – Smoking
All smoking, including environmental smoking exposure, must be avoided. Smoking is a strong and independent risk factor for heart disease.
For more information, visit:
https://www.bhf.org.uk/heart-health/risk-factors/smoking

Prevention of future heart disease – Diet
A healthy diet reduces the risk of heart disease. Calorie intake should be limited to the amount of energy needed to maintain (or obtain) a healthy weight—that is, a BMI of <25 kg/m^2.
For more information, visit:
https://www.bhf.org.uk/heart-health/preventing-heart-disease/healthy-eating

Prevention of future heart disease – Exercise
Keeping active is important. You should exercise for 30 minutes ≥3 times a week.
For more information, visit:
https://www.bhf.org.uk/heart-health/preventing-heart-disease/staying-active

Prevention of future heart disease – Weight
Weight reduction (if overweight) is recommended in order to achieve favourable effects on blood pressure, cholesterol, and diabetes control.
For more information, visit:
https://www.bhf.org.uk/heart-health/preventing-heart-disease/managing-your-weight

Prevention of future heart disease – Cholesterol
Aim for a normal cholesterol level. Please have your GP check your cholesterol and modify your medication until this is achieved.
For more information, visit:
https://www.bhf.org.uk/heart-health/risk-factors/high-cholesterol

Prevention of future heart disease – High blood pressure

Your blood pressure should be within the range of 130–139/80–85 mmHg – please have your GP check your blood pressure and modify your medication until this is achieved.
For more information, visit:
https://www.bhf.org.uk/heart-health/risk-factors/high-blood-pressure

Prevention of future heart disease – Diabetes
A healthy, low-sugar diet and regular exercise will help.
For more information, visit:
https://www.bhf.org.uk/heart-health/risk-factors/diabetes

Prevention of future heart disease – Stress
If you suffer from anxiety or depression, psychotherapy, medication and collaborative care are available through the primary care service and can be offered to you by your GP.
For more information, visit
https://www.bhf.org.uk/heart-health/preventing-heart-disease/stress

https://www.bhf.org.uk/heart-health/preventing-heart-disease/heart-and-mental-health
