# Supplementary material for: Endotyping-informed therapy for patients with chest pain and no obstructive coronary artery disease: a randomized trial
Source: Nat Med. 2025 Nov 10;32(1):332–41. doi: 10.1038/s41591-025-04044-4 (PMC12823439; doi:10.1038/s41591-025-04044-4)
Supplement: Supplementary file 2 — Reporting Summary [file 41591_2025_4044_MOESM2_ESM.pdf]

nature research

Corresponding author(s): Colin Berry BSc MBChB PhD

Last updated by author(s): Sep 18, 2025

Reporting Summary

Nature Research wishes to improve the reproducibility of the work that we publish. This form provides structure for consistency and transparency in reporting. For further information on Nature Research policies, see our Editorial Policies and the Editorial Policy Checklist.

Statistics

For all statistical analyses, confirm that the following items are present in the figure legend, table legend, main text, or Methods section.

n/a	Confirmed	
☐	☒	The exact sample size (*n*) for each experimental group/condition, given as a discrete number and unit of measurement
☐	☒	A statement on whether measurements were taken from distinct samples or whether the same sample was measured repeatedly
☐	☒	The statistical test(s) used AND whether they are one- or two-sided<br>*Only common tests should be described solely by name; describe more complex techniques in the Methods section.*
☐	☒	A description of all covariates tested
☐	☒	A description of any assumptions or corrections, such as tests of normality and adjustment for multiple comparisons
☐	☒	A full description of the statistical parameters including central tendency (e.g. means) or other basic estimates (e.g. regression coefficient) AND variation (e.g. standard deviation) or associated estimates of uncertainty (e.g. confidence intervals)
☐	☒	For null hypothesis testing, the test statistic (e.g. *F*, *t*, *r*) with confidence intervals, effect sizes, degrees of freedom and *P* value noted<br>*Give P values as exact values whenever suitable.*
☒	☐	For Bayesian analysis, information on the choice of priors and Markov chain Monte Carlo settings
☒	☐	For hierarchical and complex designs, identification of the appropriate level for tests and full reporting of outcomes
☒	☐	Estimates of effect sizes (e.g. Cohen's *d*, Pearson's *r*), indicating how they were calculated

Our web collection on statistics for biologists contains articles on many of the points above.

Software and code

Policy information about availability of computer code

Data collection	eEectronic case report form (CRF) developed by programmers in the Robertson Centre for Biostatistics. The eCRF served as a central information repository with restricted access based on centrally administered user rights determined by the chief investigator and coordinated by the Project Management team. The eCRF was developed in line with the protocol. Any changes to the eCRF required sponsor approval. Core laboratory analyses of source data were submitted by site research staff and uploaded directly to the Clinical Trials Unit (CTU) central server. These files were subject to quality assurance procedures administered by data management staff in the CTU.
Data analysis	R for Windows v4.5.1 (R Core Team (2021). R: A language and environment for statistical computing. R Foundation for Statistical Computing, Vienna, Austria. URL https://www.R-project.org/.)<br><br>Packages<br>- RODBC: Ripley B, Lapsley M (2023). _RODBC: ODBC Database Access_. R package version 1.3-23, <https://CRAN.R-project.org/package=RODBC>.<br>- eq5d: Morton F, Nijjar JS (2025). _eq5d: Methods for Analysing 'EQ-5D' Data and Calculating 'EQ-5D' Index Scores_. R package version 0.15.7, <https://CRAN.R-project.org/package=eq5d>.<br>- readxl: Wickham H, Bryan J (2025). _readxl: Read Excel Files_. R package version 1.4.5, <https://CRAN.R-project.org/package=readxl>.<br>- dplyr: Wickham H, François R, Henry L, Müller K, Vaughan D (2023). _dplyr: A Grammar of Data Manipulation_. R package version 1.1.4, <https://CRAN.R-project.org/package=dplyr>.<br>- ggplot2: H. Wickham. ggplot2: Elegant Graphics for Data Analysis. Springer-Verlag New York, 2016.<br>-tidyr survival car QRISK3- tidyr: Wickham H, Vaughan D, Girlich M (2024). _tidyr: Tidy Messy Data_. doi:10.32614/CRAN.package.tidyr <https://doi.org/10.32614/CRAN.package.tidyr>, R package version 1.3.1, <https://CRAN.R-project.org/package=tidyr>.<br>- survival: Therneau T (2024). _A Package for Survival Analysis in R_. R package version 3.8-3, <https://CRAN.R-project.org/package=survival>. Terry M. Therneau, Patricia M. Grambsch (2000). _Modeling Survival Data: Extending the Cox Model_. Springer, New York. ISBN

0-387-98784-3.
- car: Fox J, Weisberg S (2019). _An R Companion to Applied Regression_, Third edition. Sage, Thousand Oaks CA. <https://www.john-fox.ca/Companion/>.
- QRISK3: Li Y, Sperrin M, Ltd. C, van Staa TP (2023). _QRISK3: 10-Year Cardiovascular Disease Risk Calculator (QRISK3 2017)_. doi:10.32614/CRAN.package.QRISK3 <https://doi.org/10.32614/CRAN.package.QRISK3>, R package version 0.6.0, <https://CRAN.R-project.org/package=QRISK3>.

For manuscripts utilizing custom algorithms or software that are central to the research but not yet described in published literature, software must be made available to editors and reviewers. We strongly encourage code deposition in a community repository (e.g. GitHub). See the Nature Research guidelines for submitting code & software for further information.

Data

Policy information about availability of data

All manuscripts must include a data availability statement. This statement should provide the following information, where applicable:

- Accession codes, unique identifiers, or web links for publicly available datasets
- A list of figures that have associated raw data
- A description of any restrictions on data availability

Data were prospectively recorded in an electronic case database that was custom-developed by data managers based in the Robertson Centre for Biostatistics, University of Glasgow. The database had controlled access which was customized according to the roles (and blinding status) of the designations of the individual members of staff.

Code Availability
The statistical code is available online in Github: https://github.com/RobertsonCentre/CorCMR

Anonymised study data will be available on reasonable request by contacting the corresponding author (CB) via the Robertson Centre of Biostatistics. Please allow up to 1 week for a response. Professor Colin Berry, British Heart Foundation Glasgow Cardiovascular Research Centre, School of Cardiovascular and Metabolic Health, 126 University Place, University of Glasgow, Glasgow, G12 8TA, Scotland, UK. Telephone: +44 (0) 141 330 1671 or +44 (0) 141 951 5180. Fax +44 (0) 141 330 6794. Email: colin.berry@glasgow.ac.uk

Field-specific reporting

Please select the one below that is the best fit for your research. If you are not sure, read the appropriate sections before making your selection.

☒ Life sciences ☐ Behavioural & social sciences ☐ Ecological, evolutionary & environmental sciences

For a reference copy of the document with all sections, see nature.com/documents/nr-reporting-summary-flat.pdf

Life sciences study design

All studies must disclose on these points even when the disclosure is negative.

Sample size

A pre determined sample size calculation was devised by biostatistician co-authors.
The intention-to-treat analysis was the between-group comparison of the reclassification rate using logistic regression, adjusted for baseline factors associated with the likelihood of reclassification of the initial diagnosis with a sample size of 250, the 95% confidence interval of the estimate should have a width of no more than ±6.2%. This should be sufficiently precise to inform the utility of the test

The sample size was determined based on the power to detect a clinically relevant difference in the SAQ summary score. If six month outcomes could be obtained from 200 patients, the trial would have 80% power to detect a mean between-group difference in SAQ summary score of 0.40 standard deviation (SD) units. This is a small difference but we anticipated that not all patients would have their therapy changed as a result of disclosure. Using the myocardial blood flow data for the control (non-disclosure) group, we carried out focused analyses of the sub-group of patients whose therapy might have been altered based on abnormal results. For example, if therapy would be altered in 50% of patients, the study would have 80% power to detect a difference in SAQ score of 0.57 SD units for these patients; if therapy is altered in 30% of patients, there would be 80% power to detect a between-group difference of 0.74 SD units. Allowing for 20% loss-to-follow-up, 250 participants were needed to undergo stress perfusion CMR imaging.

Data exclusions

The exclusion criteria were:
(1) obstructive coronary artery disease i.e. a stenosis >70% in a single segment or 50 - 70% in two adjacent segments in a coronary artery >2.5 mm, or fractional flow reserve ≤0.80;
(2) Coronary revascularization by percutaneous coronary intervention or coronary artery bypass graft surgery following the index angiogram;
(3) Prior coronary artery bypass surgery;
(4) An alternative diagnosis that would explain the angina e.g. anemia, aortic stenosis, hypertrophic cardiomyopathy;
(5) Contra-indication to contrast-enhanced CMR e.g. estimated glomerular filtration rate < 30mL/min/1.73m2;
(6) Contra-indication to intravenous adenosine, i.e. severe asthma; long QT syndrome; second- or third-degree atrio-ventricular block and sick sinus syndrome;
(7) Lack of informed consent

Replication

N/A

Randomization

Randomization 1:1

Blinding

Study participants were blind to treatment group and CMR imaging results.

Blinding

To minimize bias, randomization was performed before the CMR scan and the participants, radiology technologists and healthcare staff responsible for clinical care were not informed of the randomized group allocation or myocardial blood flow quantified by CMR imaging and were therefore blinded. A standard CMR report was provided for all participants. The report described cardiac mass and function, and clinically significant prognostic findings, e.g. lung mass, but not the results of myocardial blood flow imaging.

The imaging cardiologist (C.Be) who reported the CMR scan was blind to the randomized group. The clinical outcome assessors were blinded to randomized group allocation.

The effectiveness of blinding was prospectively recorded in the electronic case report form.

Reporting for specific materials, systems and methods

We require information from authors about some types of materials, experimental systems and methods used in many studies. Here, indicate whether each material, system or method listed is relevant to your study. If you are not sure if a list item applies to your research, read the appropriate section before selecting a response.

Materials & experimental systems

n/a	Involved in the study	
☒	☐	Antibodies
☒	☐	Eukaryotic cell lines
☒	☐	Palaeontology and archaeology
☒	☐	Animals and other organisms
☐	☒	Human research participants
☐	☒	Clinical data
☒	☐	Dual use research of concern

Methods

n/a	Involved in the study	
☒	☐	ChIP-seq
☒	☐	Flow cytometry
☒	☐	MRI-based neuroimaging

Human research participants

Policy information about studies involving human research participants

Population characteristics

Potential participants were prospectively identified by having undergone invasive coronary angiography within 3-months, with no obstructive coronary arteries identified by angiography, and written informed consent [16]. The indication for invasive coronary angiography was 'suspected angina'. The Rose angina questionnaire [17] was used to assess symptoms at baseline and define them as typical, atypical, or non-anginal.

Sex was considered in the study design and biological sex of the participants was determined by self-reporting.

Recruitment

This study involved a prospective, multicenter, screening and recruitment in individuals.

Electronic health records for outpatients referred for assessment of possible coronary artery disease by invasive angiography at three hospitals in Scotland (National Health Service (NHS) Golden Jubilee National Hospital, University Hospital Hairmyres, and University Hospital Ayr) were screened prospectively.

A screening log was prospectively completed. The reasons for being ineligible, including lack of inclusion criteria and/or presence of exclusion criteria, were recorded. There were no selection or exclusion criteria for adults by age, sex, race or ethnicity.

Eligibility criteria

The inclusion criteria were:
(1) age ≥18 years;
(2) symptoms of angina or angina-equivalent informed by the Rose Angina questionnaire;
(3) coronary angiography ≤3 months with a plan for medical management.

The exclusion criteria were:
(1) obstructive coronary artery disease i.e. a stenosis >70% in a single segment or 50 - 70% in two adjacent segments in a coronary artery >2.5 mm, or fractional flow reserve ≤0.80;
(2) Coronary revascularization by percutaneous coronary intervention or coronary artery bypass graft surgery following the index angiogram;
(3) Prior coronary artery bypass surgery;
(4) An alternative diagnosis that would explain the angina e.g. anemia, aortic stenosis, hypertrophic cardiomyopathy;
(5) Contra-indication to contrast-enhanced CMR e.g. estimated glomerular filtration rate < 30mL/min/1.73m2;
(6) Contra-indication to intravenous adenosine, i.e. severe asthma; long QT syndrome; second- or third-degree atrio-ventricular block and sick sinus syndrome;
(7) Lack of informed consent.

The Study Information Sheet and Consent form were provided to potentially eligible patients after the standard care coronary angiogram. Patients were invited to participate after the standard care diagnosis had been assigned. Patients who

provided written informed consent attended a reference center (NHS Golden Jubilee hospital) for noninvasive endotyping by stress/rest CMR imaging.

Ethics oversight

The study was approved by the UK National Research Ethics Service (Reference 20/WS/0159).

Note that full information on the approval of the study protocol must also be provided in the manuscript.

Clinical data

Policy information about clinical studies

All manuscripts should comply with the ICMJE guidelines for publication of clinical research and a completed CONSORT checklist must be included with all submissions.

Clinical trial registration

ClinicalTrials.gov: NCT04900961

Study protocol

The study protocol was peer reviewed and published.

Bradley CP, Orchard V, McKinley G, Heggie R, Wu O, Good R, Watkins S, Lindsay M, Eteiba H, McGowan J, McGeoch R, Corcoran D, Kellman P, McConnachie A, Berry C. The coronary microvascular angina cardiovascular magnetic resonance imaging trial: Rationale and design. Am Heart J. 2023 Nov;265:213-224. doi: 10.1016/j.ahj.2023.08.067. Epub 2023 Aug 30. PMID: 37657593.

Data collection

The study involved three hospitals in central and west Scotland (catchment area, population 2.5 million) - Golden Jubilee National Hospital, University Hospital Hairmyres, and University Hospital Ayr). Two hundred and seventy-three patients were screened and provided informed consent between 9 February 2021 and 18 August 2023 and two hundred and fifty of these patients attended for CMR imaging and were randomized before the scan. Follow-up was continued for 12-months and 100% of the participants complied with the follow-up assessments. There was 0 loss to followup.

Outcomes

The study design involved two parts; first, a diagnostic study of coronary endotypes and second, a nested, randomized, controlled trial of the effects of inclusion of the myocardial blood flow results to inform the final diagnosis.

Primary outcome.

The primary outcome was the between-group difference in the reclassification rate of the initial diagnosis based on coronary angiography versus the final diagnosis after noninvasive endotyping.

Secondary outcomes

Prespecified secondary outcomes included the change in health status as measured by the Seattle Angina Questionnaire (SAQ) summary score and the 5-level EQ-5D questionnaire measured at six and twelve months.

Statistical analyses

Primary outcomes of the observational diagnostic study and the randomized trial

For the diagnostic study, we assessed the reclassification rate of the initial diagnosis based on coronary angiography versus the final diagnosis after noninvasive endotyping. This was reported as a percentage with a 95% confidence interval.

For the randomized trial, the change in SAQ Summary Score was compared between randomized groups using linear regression, adjusting for baseline SAQ SS and factors used in the minimization algorithm; age was additionally included as a covariate to reduce residual variation. The intervention effect estimate (adjusted between-group mean difference) was reported with a 95% confidence interval and p-value.

In a post-hoc analysis, for the primary outcome, we tested the interaction between the randomized intervention effect, and whether the diagnosis based on coronary angiography differed from the diagnosis based on noninvasive endotyping. The interaction p-value is reported, along with the within-subgroup intervention effect estimates.

Secondary outcomes

Secondary outcomes in the randomized trial were analysed in the same way as the primary outcome. Residual distributions were examined visually, and standard transformations were applied where necessary to improve model fit. Comparisons of other secondary and exploratory outcome variables were done using Fisher tests (categorical outcomes) or the Mann-Whitney U test (continuous outcomes) where appropriate.

In preparing this manuscript we have followed the Sex and Gender Equity in Research: rationale for the SAGER guidelines and recommended use. https://researchintegrityjournal.biomedcentral.com/articles/10.1186/s41073-016-0007-6
