# Supplementary material for: Endotyping-informed therapy for patients with chest pain and no obstructive coronary artery disease: a randomized trial
Source: Nat Med. 2025 Nov 10;32(1):332–41. doi: 10.1038/s41591-025-04044-4 (PMC12823439; doi:10.1038/s41591-025-04044-4)
Supplement: Supplementary file 4 — Trial information. [file 41591_2025_4044_MOESM4_ESM.pdf]

Supplementary Note

Trial information: author designations, role, blinding status and affiliation

Research cardiologist (unblinded)

Conor P. Bradley[1,2] MBChB

Imaging cardiologist (blinded)

Christina Tiller[4] MD/PhD, Colin Berry[1,2] BSc/MBChB/PhD

MRI Technologists (blinded)

Vanessa Orchard[2] MSc

Imaging and signal processing (blinded)

Peter Kellman[7] PhD

Clinical and Interventional Cardiologists (blinded)

Daniel Ang[1,2] MBChB/PhD, Richard Brogan[2] MD, David Carrick[5] MBChB/PhD, Damien Collison[2] MB BCh/MD, Hany Eteiba[2] MBChB, Angie Ghattas[2] MD, Richard Good[1,2] MD, Mitchell Lindsay[2] MD, Peter McCartney[2] MBChB/PhD, James McGowan[6] MD, Ross McGeoch[5] MD, Keith Robertson MBChB/PhD, Paul Rocchiccioli[2] MBChB/PhD, Aadil Shaukat[2] MBChB, Stuart Watkins[2] MD[1,2]

Research coordinators (nurse) (blinded)

Pamela Gildea[2] BN, Maria Petty[2] BN

Clinical adjudication (blinded)

Christina Tiller[4] MD/PhD, Andrew Morrow[1,2] MBChB/PhD, Robert Sykes[1,2] BMedSci(Hons)/MBChB/MRes

Biostatisticians (blinded)

Gemma McKinley[3] BSc, Beth Stanley[3] MSc, Alex McConnachie[3] PhD

Site and Principal Investigators

University Hospital Hairmyres, East Kilbride, United Kingdom

Ross McGeoch[5] MD

University Hospital Ayr, Ayr, United Kingdom
James McGowan[6] MD

Golden Jubilee University National Hospital, Clydebank, United Kingdom
Colin Berry[1,2] BSc/MBChB/PhD (Chief Investigator)

Sponsor

NHS Golden Jubilee, Clydebank, United Kingdom

Institutions

[1]British Heart Foundation Glasgow Cardiovascular Research Centre, University of Glasgow, Glasgow, United Kingdom

[2]Golden Jubilee University National Hospital, Clydebank, United Kingdom

[3]Robertson Centre for Biostatistics, University of Glasgow, Scotland, United Kingdom

[4]Internal Medicine III, Cardiology & Angiology, Innsbruck Medical University, Innsbruck, Austria

[5]Department of Cardiology, University Hospital Hairmyres, East Kilbride, United Kingdom

[6] Department of Cardiology, University Hospital Ayr, Ayr, United Kingdom

[7]National Heart, Lung and Blood Institute, National Institutes of Health, Bethesda, MD, United States
